# GB Virus C infection in Patients With HIV/Hepatitis C Virus Coinfection: Improvement of the Liver Function in Chronic Hepatitis C

**DOI:** 10.5812/hepatmon.14169

**Published:** 2014-03-03

**Authors:** Yue Feng, Li Liu, Yue-Mei Feng, Wenhua Zhao, Zheng Li, A-Mei Zhang, Yuzhu Song, Xueshan Xia

**Affiliations:** 1 Faculty of Life Science and Technology, Kunming University of Science and Technology, Kunming, China; 2 Research Institute of Nutrition and Food Sciences, Kunming Medical University, Kunming, China; 3 The Key Laboratory of Tropical and Subtropical Animal Disease, Kunming, China; 4 Department of Clinical Laboratory, The First People’s Hospital of Yunnan Province, Kunming, China

**Keywords:** GB virus C, Coinfection, HIV, Hepatitis C virus, Liver Function Tests

## Abstract

**Background:**

Previous studies in patients with hepatitis C virus (HCV)/HIV coinfection have shown that the presence of GBV-C is associated with significantly less compensated and decompensated cirrhosis, and an improvement in cirrhosis-free survival.

**Objectives:**

This study aimed to describe the effect of GBV-C in patients with chronic hepatitis C and HIV coinfection.

**Patients and Methods:**

We retrospectively studied 105 injecting drug users with chronic hepatitis C and HIV coinfection and 72 patients with chronic HCV mono-infections. Plasma samples were tested for GBV-C RNA with primers to the 5’untranslated region gene. HIV and HCV viral load, CD4^+^ and CD8^+^ cell count, and the levels of alanine aminotransferase (ALT) and aspartate aminotransferase (AST) were tested in all patients.

**Results:**

GBV-C RNA was identified in 34 (32.38%) of the patients with HIV/HCV coinfection, and in 24 (33.33%) of the patients with HCV mono-infection. GBV-C infection was associated with significantly lower ALT and AST levels in patients with chronic hepatitis C and HIV coinfection, but not in those HCV mono-infections. The presence of GBV-C infection was not correlated with CD4^+^ and CD8^+^ cell count, gender, age, HIV load, HCV load, and HCV genotype.

**Conclusions:**

This study found that GBV-C infection has a high frequency among injecting drug users with HIV/HCV coinfection and HCV mono-infection in Yunnan, China. In patients with chronic hepatitis C and HIV coinfection, GBV-C RNA was associated with significantly lower ALT and AST levels, suggesting a beneficial effect of GBV-C infection on chronic hepatitis C.

## 1. Background

GB virus C is classified as pegivirus of the family flaviviridae, based on its genome organization and phylogenetic analysis. GBV-C belongs to positive sense single stranded RNA virus with about 9.4 kb nucleotides. It is mainly transmitted through parenteral exposure to blood or blood products, sexual contact, and less frequently through mother-to-child transmission (1). Due to the shared transmission modes with HIV and hepatitis C virus (HCV), coinfection with GBV-C is common among people infected with HIV and/or HCV. Approximately, 10%-25% of patients with chronic hepatitis C and 14%–36% of injecting drug users (IDUs) seropositive for HIV, show the evidence of GBV-C coinfection (2, 3). The higher GBV-C triple infection rate of 30%–36% among individuals with HIV/HCV coinfection has been reported by recent investigations (4, 5). GBV-C is a nonpathogenic virus. Many studies have confirmed that GBV-C did not cause liver or any other human diseases (6, 7). However, its coinfection with HIV may produce some favorable outcomes, with a lower mortality rate, slower disease progression, and longer survival term (8, 9). Nonetheless, not all studies undoubtedly support its beneficial consequences (10, 11).

GBV-C infection is universal in IDUs with HCV mono- infection and HIV/HCV coinfection and has no effect on the course of liver disease in HCV mono-infection (10-12). Conversely, HIV/HCV coinfection speeds up HCV-related hepatic fibrosis (13). Recently, some studies have found that GBV-C exerted a beneficial effect on patients with HCV-related liver cirrhosis with lower compensated and decompensated cirrhosis, and longer cirrhosis-free survival in subjects with HIV/HCV coinfection (6).

## 2. Objectives

To describe the effect of GBV-C in chronic hepatitis C patients with HIV/HCV coinfection, this study investigates the association between GBV-C, HIV and chronic hepatitis C in a cohort of IDUs with HIV/HCV coinfection and HCV mono- infection.

## 3. Patients and Methods

### 3.1. Study Population

From June 2008 to August 2011, a total of 177 HIV or HCV seropositive IDUs were retrospectively studied in drug rehabilitation center at Dali, a famous tourism city in Yunnan province, China. Recruited subjects include 105 patients with HIV and HCV coinfection and 72 patients with HCV mono- infection. The infection was confirmed by HIV and HCV RNA quantitative detection using the Roche Cobas Amplicor 2.0 assay kit (Roche Diagnostic GmbH, Germany). The clinical parameters of disease progression, including the ALT and AST levels, and CD4^+^ and CD8^+^ lymphocyte count were determined at sampling time; demographic data were recorded and subsequently analyzed. This study was approved by the Institutional Ethics Committee. To be eligible for this study, the diagnosis of chronic HCV infection was confirmed by the presence of anti-HCV antibodies and more than 6 months HCV-RNA in the collected sera. The patients with chronic HCV hepatitis did not receive treatment for HCV. Clinical and biochemical parameters, computed tomography, and hepatitis questionnaire were reviewed to determine the extent of liver disease in the absence of liver biopsy. The exclusion criteria were: 1) patients without clinical information; 2) liver cirrhosis and liver cancer; 3) significant alcohol consumption and/or alcoholic liver disease; 4) coinfection with hepatitis B (positive HBs antigen); 5) anti-HCV antibody negative with HCV RNA positive.

### 3.2. GBV-C Virology Analysis

The stored plasma specimens from patients with HIV/HCV coinfection and HCV mono-infection were tested for GBV-C RNA. In detail, viral RNA was extracted with high pure viral RNA Kit (Roche Diagnostic GmbH, Germany). The target gene was amplified using one-step RT-PCR system (TaKaRa, Japan) combined with nest-PCR as previously described (14). HCV genotype was determined in our previous reports (15).

### 3.3. Statistical Analysis

Student t test was used to determine the differences of age, ALT, AST, CD4^+^ and CD8^+^ cell count, and HIV and HCV viral load between the GBV-C-infected and uninfected subjects. Chi-square tests were used to compare categorical variables such as gender, HAART treatment, and HCV genotype. P value < 0.05 was considered statistically significant.

## 4. Results

### 4.1. GBV-C Infection Among Injecting Drug Users With HIV-1/HCV Coinfection 

Among 105 patients with HIV/HCV coinfection, 34 cases were detected to have positive GBV-C RNA results. It accounted for 32.38% of GBV-C infection rate among patients with HIV and HCV coinfection. Comparatively, from 72 patients with HCV mono-infection, 24 cases had positive GBV-C RNA results, which meant the GBV-C infection rate of 33.33%. There was no significant difference with regard to GBV-C infection rate between patients with HIV/HCV coinfection and those with HCV mono-infection. Regarding the demographic data analysis, no significant difference in gender and age was found between patients with HIV/HCV coinfection and HCV mono-infection. It was shown that the gender and age had no effect on GBV-C infection neither in the HIV/HCV coinfected group nor in the HCV mono-infected group. Among the 105 individuals with HIV-1/HCV coinfection, 75 (71.43%) subjects were receiving highly active antiretroviral therapy (HAART) treatment among which 12 (35.29%) subjects had positive results for GBV-C RNA. This GBV-C infection rate was not significantly different from the rate in untreated patients. HCV genotyping was conducted in a majority of IDUs in our previous reports (15). They were determined to be infected with HCV strains classified as genotype 1, 3, and 6, respectively. HCV genotype was available for 75 out of the 105 patients with HIV/HCV coinfection. Furthermore, from 28 patients with positive GBV-C results, 4 (14.28%) were HCV genotype 1, 17 (60.72%) were genotype 3, and 7 (25%) were genotype 6 (Table 1). From 47 individuals with negative GBV-C results, 8 (14.02%) were HCV genotype 1, 33 (70.21%) were genotype 3, and 6 (12.55%) were genotype 6 (Table 1). However, there was no statistically significant difference between the patients with positive and negative GBV-C results (P = 0.410). The frequency of each HCV genotype in patients with HCV mono-infection is shown in Table 2. Similarly, there was also no significant difference regarding HCV genotype through comparative analysis between groups (P = 0.086).

**Table 1.** Differences Between Injecting Drug Users With HIV-1 and HCV Coinfection With Regard to Positive and Negative Results for GBV-C Infection ^a^, ^b^

Variable	GBV-C-infected	GBV-C-uninfected	P Value
**Patients**	34 (32.38)	71 (67.62)	NA
**Gender**			0.781 ^c^
Male	27	58	
Female	7	13	
**Age, y**	31.25 ± 0.64	32.22 ± 0.63	0.366 ^d^
**ALT, IU/L**	52.83 ± 5.09	75.98 ± 9.905	0.041 ^d^
**AST, IU/L**	18.69 ± 3.36	40.26 ± 9.77	0.043 ^d^
**CD4^+^Count, cells/UL**	414.8 ± 65.95	345.6 ± 21.43	0.393 ^d^
**CD8^+^Count, cells/UL**	1124 ± 182.10	1007 ± 63.80	0.554 ^d^
**Use of HAART**	12 (35.29)	22 (30.98)	0.659 ^c^
**HIV-1 RNA, Log_10_ copies/mL**	3.80 ± 0.14	3.93 ± 0.26	0.654 ^d^
**HCV RNA, Log_10_copies/mL**	5.60 ± 0.31	5.90 ± 0.16	0.378 ^d^
**HCV genotype**			0.410 c
1	4 (14.28)	8 (14.02)	
3	17 (60.72)	33 (70.21)	
6	7 (25.00)	6 (12.77)	

^a^ Abbreviations: ALT, alanine aminotransferase; AST,aspartate aminotransferase; HAART, highly active antiretroviral therapy; HCV, hepatitis C virus; NA, not applicable.

^b^ Data are presented in Mean ± SD or No. (%).

^c^ Person’s chi-squared test.

^d^ unpaired t-test with Welch’s correction.

**Table 2.** Differences Between Injecting Drug Users With HCV Mono-infection With Regard to Positive and Negative GBV-C-Infection ^a^

Variable	GBV-C- Infected	GBV-C-Uninfected	P Value
**Patients**	24 (33.33)	48 (66.67)	NA
**Gender**			0.514 ^b^
Male	19	28	
Female	5	20	
**Age, y**	50.56 ± 6.17	55.92 ± 5.08	0.507 ^c^
**ALT, IU/L**	67.61 ± 19.64	58.85 ± 5.98	0.677 ^c^
**AST, IU/L**	12.67 ± 5.55	9.07 ± 1.45	0.595 ^c^
**CD4^+^Count, cells/UL**	600.9 ± 91.53	523.6 ± 48.43	0.466 ^c^
**CD8^+^Count, cells/UL**	700.5 ± 133.80	682.9 ± 63.41	0.909 ^c^
**HCV RNA, Log_10_copies/mL**	5.06 ± 0.57	5.36 ± 0.36	0.647 ^c^
**HCV genotype**			0.086 b
1	9 (45.0)	8 (20.51)	
3	9 (45.0)	29 (74.36)	
6	2 (10.0)	2 (5.13)	

^a^ Data are presented in Mean ± SD or No. (%).

^b^ Person’s chi-squared test.

^c^ unpaired t-test with Welch’s correction.

**4.2. Effect of GBV-C Coinfection on HIV-1 or HCV **-**Related Disease Progression**

Based on results for GBV-C, 105 patients with HIV and HCV coinfection were divided into GBV-C infected (34 cases) and uninfected (71 cases) groups. The statistical analysis did not show any significant difference with regard to the major parameters of AIDS progression, such as CD4^+^ and CD8^+^ cell count and HIV viral load. Besides, patients with GBV-C infection seem to have a higher CD4^+^ cell count (414.8 ± 65.5 cells/μL) than patients without GBV-C infection (345.6 ± 21.43 cells/μL); however, the difference was not significant (P = 0.393). Significantly lower ALT (52.89 ± 5.09 IU/L) and AST (18.69 ± 3.36 IU/L) levels were found in patients with positive GBV-C RNA results than patients with negative GBV-C results (ALT of 75.98 ± 9.905 IU/L; AST of 40.26 ± 9.77 IU/L) with P values of 0.041 and 0.043, respectively. For patients with HCV mono-infection, the CD4^+^ and CD8^+^ cell counts were normal. There was no significant difference concerning ALT and AST levels between patients with positive and negative GBV-C results. In addition, HCV viral load in patients with mono-infection was not affected by GBV-C infection.

## 5. Discussion

In the present study, we investigated the prevalence and impact of GBV-C infection in a cohort of 105 patients with HIV/HCV coinfection and 72 patients with HCV mono-infection. We found a prevalence of 32.38% in patients with HIV/HCV coinfection and 33.33% in patients with HCV mono-infection. Our findings were consistent with the previous reports in the patients with HIV/HCV coinfection from Australia (6) and Germany (5), suggesting a similar transmission efficiency for the three viruses among high risk group. Moreover, the higher prevalence of GBV-C RNA was 33.33% in our cohort with HCV mono-infection than other studies (16, 17). This might be explained by the impact of HCV treatment on GBV-C RNA clearance. Although, the genotype distribution of HCV in patients with positive and negative GBV-C results in mono and dual HIV/HCV cases were different (Tables 1 and 2), there was no significant interaction between HCV genotype and GBV-C infection status in our multivariable analysis. It was difficult to conclude whether the HCV genotype influences the clinical outcome due to the limited number of cases in this cohort or not. Larger studies and follow-up studies are necessary, and further studies are needed to determine GBV-C genotype, GBV-C viral load, and the results of liver biopsy.

Many studies have shown that GBV-C and HIV coinfection was associated with higher CD4^+^ cell count and lower HIV load (8, 9). Unfortunately, in agree with previous reports, we did not found any significant difference in CD4^+^ cell counts and HIV load with regard to the GBV-C infection status among patients with HIV/HCV coinfection (5, 6). It might be due to the samples size of the study group, the different genotypes of GBV-C and/or HIV, and the lack of the detection of antibodies to the E2 protein of GBV-C. An important finding from our study was that the GBV-C infection was associated with significantly lower ALT and AST levels, which might have a favorable impact on chronic hepatitis C- related liver function in patients with HIV/HCV coinfection. These findings might offer direct evidence for the importance of an improved effect of GBV-C on chronic hepatitis C- related liver function. These results were consistent with the prior research that stated GBV-C infection led to reduced liver disease in HIV/HCV coinfection (5, 6, 18). Unlike our study, they showed that GBV-C RNA was associated with significantly less compensated and decompensated cirrhosis, and with improvement in cirrhosis-free survival among patients with HCV-related liver cirrhosis, which were not seen in our study (6). Similar to other reports, our study also demonstrated that GBV-C did not appear to increase liver injury in patients with chronic HCV mono-infection, as measured by the levels of ALT, AST, and HCV load (19, 20). These two observations clearly supported the beneficial effect of GBV-C RNA on the liver disease only in HIV/HCV coinfection cohort, not HCV mono-infection. Although, the mechanism might be down-regulation of lymphocyte-specific protein tyrosine kinase (LCK) gene involved in intrahepatic T-cell signaling in HIV/HCV coinfection (18), which should be further confirmed in the patients., The patients should be followed up to see the effect of GBV-C on chronic hepatitis C-related liver function in patients with HIV/HCV coinfection to further verify the outcome accurately. There were several limitations to our study including the small number of patients with HIV/HCV coinfection, no evaluation of the role of GBV-C genotypes, and lack of data concerning GBV-C antibody and GBV-C load. Further studies are essential to overcome these important limitations. In summary, GBV-C infection was common in this cohort of IDUs with HIV/HCV coinfection and HCV mono-infection, and appeared to improve the chronic hepatitis C-related liver function in patients with HIV/HCV coinfection with lower ALT and AST levels.

## References

[1] Stapleton JT, Foung S, Muerhoff AS, Bukh J, Simmonds P (2011). The GB viruses: a review and proposed classification of GBV-A, GBV-C (HGV), and GBV-D in genus Pegivirus within the family Flaviviridae.. J Gen Virol..

[2] Bhattarai N, Stapleton JT (2012). GB virus C: the good boy virus?. Trends Microbiol..

[3] Petrik J, Guella L, Wight DG, Pearson GM, Hinton J, Parker H (1998). Hepatic histology in hepatitis C virus carriers coinfected with hepatitis G virus.. Gut..

[4] Hekmat S, Mohraz M, Vahabpour R, Jam S, Bahramali G, Banifazl M (2008). Frequency and genotype of GB virus C among Iranian patients infected with HIV.. J Med Virol..

[5] Schwarze-Zander C, Blackard JT, Zheng H, Addo MM, Lin W, Robbins GK (2006). GB virus C (GBV-C) infection in hepatitis C virus (HCV)/HIV-coinfected patients receiving HCV treatment: importance of the GBV-C genotype.. J Infect Dis..

[6] Berzsenyi MD, Bowden DS, Kelly HA, Watson KM, Mijch AM, Hammond RA (2007). Reduction in hepatitis C-related liver disease associated with GB virus C in human immunodeficiency virus coinfection.. Gastroenterology..

[7] Schwarze-Zander C, Blackard JT, Rockstroh JK (2012). Role of GB virus C in modulating HIV disease.. Expert Rev Anti Infect Ther..

[8] Keyvani H, Mohammadi A, Sabouri Ghannad M, Hajabdolbaghi M (2012). The Effect of GBV-C Infection on CD4 Count and Viral Loads in Patients Infected With HIV.. Hepat Mon..

[9] Xiang J, Wunschmann S, Diekema DJ, Klinzman D, Patrick KD, George SL (2001). Effect of coinfection with GB virus C on survival among patients with HIV infection.. N Engl J Med..

[10] Bjorkman P, Flamholc L, Naucler A, Molnegren V, Wallmark E, Widell A (2004). GB virus C during the natural course of HIV-1 infection: viremia at diagnosis does not predict mortality.. AIDS..

[11] Kaye S, Howard M, Alabi A, Hansmann A, Whittle H, Schim van der Loeff M (2005). No observed effect of GB virus C coinfection on disease progression in a cohort of African woman infected with HIV-1 or HIV-2.. Clin Infect Dis..

[12] Ghanbari R, Ravanshad M, Hosseini SY, Yaghobi R, Shahzamani K (2010). Genotyping and infection rate of GBV-C among Iranian HCV- infected patients.. Hepat Mon..

[13] Lin W, Weinberg EM, Chung RT (2013). Pathogenesis of accelerated fibrosis in HIV/HCV co-infection.. J Infect Dis..

[14] Feng Y, Zhao W, Feng Y, Dai J, Li Z, Zhang X (2011). A novel genotype of GB virus C: its identification and predominance among injecting drug users in Yunnan, China.. PLoS One..

[15] Xia X, Lu L, Tee KK, Zhao W, Wu J, Yu J (2008). The unique HCV genotype distribution and the discovery of a novel subtype 6u among IDUs co-infected with HIV-1 in Yunnan, China.. J Med Virol..

[16] Hofer H, Aydin I, Neumueller-Guber S, Mueller C, Scherzer TM, Staufer K (2011). Prevalence and clinical significance of GB virus type C/hepatitis G virus coinfection in patients with chronic hepatitis C undergoing antiviral therapy.. J Viral Hepat..

[17] Berzsenyi MD, Bowden DS, Roberts SK (2005). GB virus C: insights into co-infection.. J Clin Virol..

[18] Berzsenyi MD, Woollard DJ, McLean CA, Preiss S, Perreau VM, Beard MR (2011). Down-regulation of intra-hepatic T-cell signaling associated with GB virus C in a HCV/HIV co-infected group with reduced liver disease.. J Hepatol..

[19] Pereira LM, Spinelli V, Ximenes RA, Cavalcanti MS, Melo R, Juca N (2002). Chronic hepatitis C infection: influence of the viral load, genotypes, and GBV-C/HGV coinfection on the severity of the disease in a Brazilian population.. J Med Virol..

[20] Tan D, Matsumoto A, Conry-Cantilena C, Melpolder JC, Shih JW, Leuther M (1999). Analysis of hepatitis G virus (HGV) RNA, antibody to HGV envelope protein, and risk factors for blood donors coinfected with HGV and hepatitis C virus.. J Infect Dis..

